# Iodine status of 8 to 10 years old children within 20 years following compulsory salt iodization policy in Shanghai, China

**DOI:** 10.1186/s12937-019-0491-x

**Published:** 2019-11-02

**Authors:** Zhengyuan Wang, Jiajie Zang, Zhehuan Shi, Zhenni Zhu, Jun Song, Shurong Zou, Wei Jin, Xiaodong Jia, Changyi Guo, Shoujun Liu

**Affiliations:** 1 Division of Health Risk Factors Monitoring and Control, Shanghai Municipal Center for Disease Control and Prevention, Shanghai, 200336 China; 2 Laboratory of Hygienic Inspection, Shanghai Municipal Center for Disease Control and Prevention, Shanghai, 200336 China; 3 General Office, Shanghai Municipal Center for Disease Control and Prevention, Shanghai, 200336 China; 4 Institute of Iodine Deficiency Disorders, Center for Endemic Disease Control, Chinese Center for Disease Control and Prevention, Harbin Medical University, Harbin, 150081 Heilongjiang China

**Keywords:** Salt iodine concentration, Urinary iodine concentration, Thyroid volume, Goiter, Shanghai

## Abstract

**Background:**

In 1996, Shanghai implemented universal salt iodization and has became the last provincial unit in China to carry out this intervention. In this study, we summarized achievements in past 20 years, to provide suggestions and evidence for the next stage of iodine supplementation.

**Methods:**

This study summarized and analyzed monitoring data of children from 1997, 1999, 2005, 2011, 2014, and 2017 in Shanghai. In each monitoring year, 30 streets or towns were selected using the probability-proportional-to-size sampling technique. One primary school was selected from each street or town by a simple random sampling technique. From each school, 40 children aged 8 to 10 years were randomly selected. The number of children was divided equally by sex and age.

**Results:**

In 1997, 1999, 2005, 2011, 2014, and 2017, median urinary iodine (MUI) was 227.5 μg/L, 214.3 μg/L, 198.1 μg/L, 181.6 μg/L, 171.4 μg/L, and 183.0 μg/L, goiter rate was 3.07, 0.40, 0.08, 0.08, 0.86, and 1.90%, and median thyroid volume (MTvol) was 2.9 mL, 1.2 mL, 2.4 mL, 1.0 mL, 1.8 mL, and 2.8 mL, respectively. There was a linear correlation between goiter rate and median thyroid volume (MTvol) (*r* = 0.95, *P* = 0.014). Household salt iodine concentration (SIC) was dropping every monitoring (*P* < 0.05). There was a significant difference among different household SIC groups in MUI in 1999 and 2017, and in MTvol in 1999 (*P* < 0.05). No significant differences were detected in the other years.

**Conclusions:**

In Shanghai, the iodine status of 8 to 10 years old children is adequate. Household SIC have little effect on iodine status of children. Future studies should analyze the dietary sources of iodine, especially from pre-packaged and prepared-away-from-home foods or meals. The regular monitoring of iodine status is important to human health.

## Background

Iodine is indispensable for thyroid hormone synthesis. Long-term iodine deficiency (ID) can lead to iodine deficiency disorders (IDD), such as endemic goiter and cretinism, which may have irreversible effects on the physical and mental development of individuals [1–3]. ID used to be a major public health problem worldwide. According to a 2001 report by the World Health Organization (WHO), approximately 285 million of school-age children with ID were under risk of IDD [4]. Given the high prevalence of ID and IDD in China, universal salt iodization (USI) was implemented in 1994 to prevent and control for IDD [5].

Shanghai is a coastal city. The first iodine status monitoring in Shanghai in 1995 showed that goiter rate was low (i.e. 1.57% among urban students aged 9 to 13 years and 3.71% among adults), however, median urinary iodine (MUI) was 69.62 μg/L among urban students aged 9 to 13 years [6], suggesting that the overall iodine nutritional status was low. Therefore, USI has been carried out in Shanghai since 1996. As a result, the iodine nutritional status of Shanghai residents is often assessed separately.

Since the implementation of USI, Shanghai has carried out a number of nutritional assessments. Studies from the International Council for Control of Iodine Deficiency Disorders (ICCIDD), United Nations Children’s Fund (UNICEF), and WHO have revealed that the iodine concentration of a spot urine sample from a child can be used to estimate the iodine status of the population [7]. Thyroid volume (Tvol) of children is the most sensitive morphological index of ID, and goiter is the typical manifestation of thyroid dysfunction [8]. The population we monitored in this study consisted of school-age children between the ages of 8 and 10. The main indicators were urinary iodine concentration (UIC), household salt iodine concentration (SIC), and Tvol. Standard household SIC was adjusted three times according to the monitoring results. In Shanghai, household SIC was 20 to 60 mg/kg in 1996, 20 to 50 mg/kg in 2000, and 21 to 39 mg/kg in 2012 [9]. We observed that UIC and Tvol varied, and the changes were not completely consistent with the changes in household SIC. The objectives of this study were to 1) analyze data from 1997, 1999, 2005, 2011, 2014, and 2017, 2) estimate the iodine nutritional status of Shanghai residents, 3) summarize the achievements in the prevention and treatment of IDD in the past 20 years, and 4) provide suggestions for the next stages of iodine supplementation.

## Methods

### Population and study design

Data from the National IDD Monitoring Program were obtained for 1997, 1999, 2005, 2011, 2014, and 2017. In each year, a probability-proportional-to-size sampling technique was used to select 30 sampling units. In each sampling unit, one primary school was randomly selected. From each selected school, 40 children between the ages of 8 and 10 were randomly recruited. Tvol and household SIC were measured in all students. UIC was measured from urine samples of 12–16 children. When the selected school did not have sufficient participants, the school nearest to the selected one was included. Household SIC was not evaluated in 1997.

The medical ethics committee of the Center for Endemic Disease Control at Harbin Medical University approved the study. Written informed consent was obtained from all parents or guardians.

### Tvol measurement

Tvol was measured using a portable ultrasound machine by professionals who were trained and qualified by national experts. Thyroid lobe volume was calculated according to the following formula, V (mL) = 0.479 × d × w × 1 (mm)/1000, where d is the depth, w is the width, and l is the length of each lobe. Tvol was calculated as the sum of the volumes of both lobes; however, the volume of the isthmus was not included [10]. According to Chinese criteria (WS 276–2007), goiter was defined as Tvol > 4.5 mL in 8-year old children, > 5.0 mL in 9-year old children, and > 6.0 mL in 10-year old children [11].

### Household salt sampling, urine sample collection and analyses

More than 100 g of household salt sample and 5 ml urine sample from each subject were collected. Household SIC and UIC were measured by titration and acid digestion [12], respectively, at the Central Laboratory of Shanghai Municipal Center for Disease Control and Prevention and 17 Districts Center for Disease Control and Prevention in Shanghai. All laboratories were required to conduct blind sample examination, and only those who pass the examination were allowed to participate in the test. In the testing process, one internal quality-control sample was tested after every 20 test samples to ensure accuracy of the test results. The blind samples and internal quality-control samples used for the analysis of salt iodine and urinary iodine concentrations were provided by the China National Iodine Deficiency Disorders Reference Laboratory which was the most authoritative salt iodine and urinary iodine concentrations testing laboratory in China. Acceptable iodized salt contained an iodine concentration of more than 20 mg/kg and less than 60 mg/kg in 1999, more than 20 mg/kg and less than 50 mg/kg in 2005, 2011, and 2014, and more than 21 mg/kg and less than 39 mg/kg in 2017. The iodine nutritional status of children was determined according to the recommended WHO/UNICEF/ICCIDD criteria: insufficient iodine intake was defined as MUI < 100 μg/L; adequate iodine intake, as MUI 100–199 μg/L; iodine intake above the requirement, as MUI 200–299 μg/L; and excessive iodine intake, as MUI ≥ 300 μg/L [4].

Household coverage with iodized salt was defined as the number of households consuming iodized salt divided by the total number of households surveyed.

### Statistical analysis

Data processing and statistical analyses were conducted with Excel (2010 Edition, Microsoft, China) and SAS (9.2 Edition, SAS Institute, Cary, NC, USA). Data that were normally distributed were expressed as mean ± SD; non-parametric data were expressed as the median (25th percentile, 75th percentile). T test was used to compare normally distributed data between two groups, one-way ANOVA was used to compare multiple groups. In pairwise comparisons, the homogeneity of variance was tested by LSD, and the inhomogeneity of variance was assessed by Tamhane’s T2 test. Mann-Whitney U test was used to compare the non-parametric data between two groups, Kruskal Wallis-one-way ANOVA test (K) was used to compare multiple groups, pairwise comparisons were evaluated by the pairwise method. The Bonferroni method was used to calibrate α. The formula was as follows: adjusted α = pre-adjusted α / number of pairwise comparisons. The correlation between goiter rate and MTvol was first observed by scatter plot to exclude outliers and check whether data conform to assumptions of linear correlation, if so then analyzed by linear correlation. *P* value < 0.05 was considered significant.

## Results

### Household SIC of 8 to 10 years old children

Household coverage with iodized salt was in the form of a drop in 2005 after the first rise. Average iodine concentrations of salt were 43.8 ± 16.3 mg/kg, 32.9 ± 4.0 mg/kg, 28.9 ± 5.8 mg/kg, 25.8 ± 9.2 mg/kg, and 24.3 ± 5.6 mg/kg in 1999, 2005, 2011, 2014, and 2017, respectively. The results showed that mean SIC (MSI) was in the range of the standard household SIC each monitoring year (Table 1).

**Table 1:** Household SIC of 8 to 10-year old children

Standard of SIC	Year	Sample size	Household coverage with iodized salt (%)	SIC (mg/kg) ($\overline{\mathrm{x}}$±s)
20–60 mg/kg	1999	1320	94.6	43.8 ± 16.3
20–50 mg/kg	2005	1223	98.6	32.9 ± 4.0
	2011	1234	92.3	28.9 ± 5.8
21–39 mg/kg	2014	1510	85.6	25.8 ± 9.2
	2017	1215	76.5	24.3 ± 5.6

Based on ANOVA results, the differences in household SIC among the years were statistically significant (*P* < 0.05). In pairwise comparisons, the differences between each two groups comparison were statistically significant (*P* < adjusted α = 0.005). Household SIC reduced over time.

### UIC of 8 to 10 years old children

**Fig. 1:**
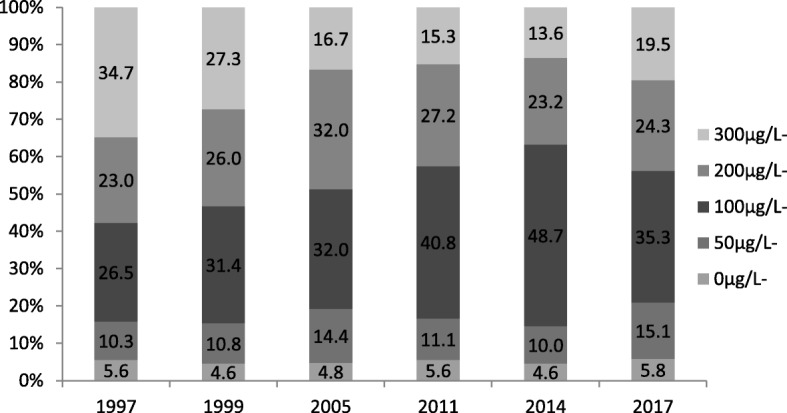
MUI distribution (μg/L) in 8 to 10 years old children among different years (median, P25, P75). Proportion of UI in 8 to 10 years old children among different years (%)

MUI of 8 to 10 years old children was 227.5 μg/L, 214.3 μg/L, 198.1 μg/L, 181.6 μg/L, 171.4 μg/L, and 183.0 μg/L in 1997, 1999, 2005, 2011, 2014, and 2017, respectively (Fig. 1 and Table 2). The percentage ratios of urinary iodine ranged between 100 and 200 μg/L were 26.5, 31.4, 32.0, 40.8, 48.7 and 35.3%, respectively (Fig. 1).

**Table 2:** MUI (μg/L) of 8- to 10-year old children (median; P25, P75)

Year	Girls				Boys				Total
	8 years	9 years	10 years	Subtotal	8 years	9 years	10 years	Subtotal	
1997	202.0 (118.0,334.0)	208.0 (114.3333.0)	238.0 (135.0,380.0)	214.0 (119.0,349.0)	235.5 (150.0,335.8)	261.5 (171.3389.8)	227.0 (127.8352.8)	241.5 (146.3364.5)	227.5 (132.0,356.5)
1999	207.1 (115.2311.7)	217.1 (125.3301.6)	194.1 (129.7283.4)	205.4 (124.2297.9)	254.1 (154.2368.4)	227.5 (150.5333.1)	205.5 (124.4300.2)	220.2 (138.4326.6)	214.3 (129.9308.4)
2005	206.1 (118.0,264.3)	199.6 (131.2278.6)	191.7 (106.6253.8)	201.1 (125.3262.5)	199.6 (110.5270.0)	207.4 (140.7274.6)	157.0 (101.9268.7)	192.7 (123.4272.3)	198.1 (124.8266.4)
2011	176.0 (112.2250.4)	180.1 (115.1255.7)	222.6 (140.1290.9)	182.0 (118.6262.0)	170.8 (125.1249.5)	188.0 (138.0,254.1)	167.1 (123.2243.5)	181.6 (132.8247.7)	181.6 (125.4254.5)
2014	168.2 (117.5236.9)	179.2 (124.4225.4)	174.0 (123.2230.5)	173.0 (122.9230.1)	171.6 (121.2262.3)	170.2 (124.9221.6)	171.0 (127.4247.5)	171.1 (124.8242.8)	171.4 (124.0,235.6)
2017	169.0 (96.0,266.5)	176.0 (122.0,253.0)	180.0 (103.4251.0)	175.0 (106.0,256.6)	184.9 (116.0,269.3)	177.5 (123.0,279.8)	202.0 (120.0,298.0)	189.0 (119.0,278.3)	183.0 (114.4267.0)

With respect to age, we obtained significant differences (*P* < 0.05) in UIC among boys in 1999; however, there were no significant differences in the other groups. With respect to sex, we observed significant differences (*P* < 0.05) in UIC in the 9-year old group in 1997 and 1999.

### Tvol of 8 to 10 years old children

Goiter rate was 3.07, 0.40, 0.08, 0.08, 0.86, and 1.90%, and median Tvol (MTvol) was 2.9 mL, 1.2 mL, 2.4 mL, 1.0 mL, 1.8 mL, and 2.8 mL in 1997, 1999, 2005, 2011, 2014, and 2017, respectively (Table 3).

**Table 3:** Tvol (mL) of 8- to 10-year old children (median; P25, P75)

Year	Girls				Boys				Total
	8 years	9 years	10 years	Subtotal	8 years	9 years	10 years	Subtotal	
1997	2.7 (2.2,3.3)	2.9 (2.4,3.7)	3.0 (2.5,3.8)	2.9 (2.3,3.6)	2.6 (2.1,3.2)	2.8 (2.3,3.5)	3.1 (2.5,4.0)	2.8 (2.3,3.5)	2.9 (2.3,3.6)
1999	1.1 (0.9,1.5)	1.1 (0.9,1.4)	1.2 (0.9,1.6)	1.1 (0.9,1.5)	1.1 (1,1.5.0)	1.2 (1,1.5.0)	1.2 (1.0,1.7)	1.2 (1,1.6.0)	1.2 (0.9,1.5)
2005	2.2 (2,2.4.0)	2.4 (2.2,2.7)	2.6 (2.3,2.9)	2.4 (2.1,2.7)	2.3 (2.0,2.5)	2.4 (2.2,2.8)	2.6 (2.3,3.0)	2.4 (2.2,2.7)	2.4 (2.2,2.7)
2011	0.9 (0.7,1.0)	1.0 (0.8,1.2)	1.1 (0.9,1.3)	1.0 (0.8,1.1)	0.9 (0.7,1.1)	1.0 (0.8,1.3)	1.1 (1,1.3.0)	1.0 (0.8,1.2)	1.0 (0.8,1.2)
2014	1.6 (0.9,2.1)	1.8 (1,2.3.0)	2.1 (1.3,2.9)	1.8 (1.0,2.4)	1.6 (1,2.2.0)	1.9 (1.1,2.5)	2.0 (1.3,2.7)	1.8 (1.1,2.5)	1.8 (1.1,2.5)
2017	2.4 (1.6,3.3)	2.8 (2.1,3.5)	3.1 (2.2,4.0)	2.8 (2.0,3.6)	2.4 (1.6,3.3)	2.7 (2.0,3.5)	3.1 (2.3,4.1)	2.7 (2.0,3.6)	2.8 (2.0,3.6)

A scatter plot between goiter rate and MTvol revealed that the data from 2015 was abnormally distributed. Correlation analysis (*r* = 0.95, *P* < 0.05) showed that there was linear relationship between goiter rate and MTvol by excluding the data in 2015.

With respect to age, we obtained significant differences (*P* < 0.05) in Tvol in the boy and girl groups of 1997, 2011, 2014, and 2017. With respect to sex, we observed significant differences in Tvol in the 8-year old group of 2005.

### Relationship between UIC, Tvol, and household SIC in 8 to 10 years old children

#### Changes in MUI with household SIC

Based on the presence of iodine in salt and median household SIC monitored over the years, cooking salt was divided into three groups: non-iodized salt group (iodine content < 5 mg/kg), low-iodine salt group (5 mg/kg ≤ iodine content < 29.6 mg/kg), and high-iodine salt group (iodine content ≥29.6 mg/kg). The differences in urinary iodine between the groups were analyzed. There were significant differences in MUI among the three groups only in 1999 and 2017 with *P* = 0.000 and *P* = 0.005, respectively. There were no statistically significant differences in other years (Table 4). Pairwise comparisons were performed between the three groups in 1999 and 2017. The adjusted *P* value < 0.05/3 = 0.017 was considered significant. The adjusted *P*-values were 1.000 and 0.004 between the non-iodized salt group and the low-iodine salt group, 0.015 and 0.316 between the non-iodized salt group and high-iodine salt group, and 0.000 and 1.000 between the low-iodine salt group and high-iodine salt group, respectively. The results showed that MUI was higher in the high-iodine salt group than in the non-iodized salt group and the low-salt iodine group in 1999. MUI was higher in the low-salt iodine group than in the non-iodized salt group in 2017.

**Table 4:** UIC (μg/L) of 8 to 10 years old children with different household SIC (Median(P25, P75))

Year	Non-iodized salt group		Low salt iodine group		High salt iodine group		χ2	*P*
	N(%)	UIC	N(%)	UIC	N(%)	UIC		
1999	70 (5.4)	181.7 (119.0,245.1)	219 (17.0)	164.3 (98.1258.4)	999 (77.6)	228.5 (145.0,322.0)	35.4	0.000
2005	7 (1.6)	117.8 (71.8214.5)	76 (17.4)	200.0 (143.7261.0)	354 (81.0)	198.3 (124.0,268.8)	4.0	0.133
2011	37 (10.3)	176.4 (125.8259.2)	184 (51.1)	190.8 (130.6262.5)	139 (38.6)	170.5 (116.5251.7)	2.8	0.244
2014	217 (14.4)	171.1 (124.1235.5)	1038 (68.7)	171.0 (119.1239.4)	255 (16.9)	176.2 (133.3226.4)	1.3	0.514
2017	285 (23.5)	166.0 (98.3239.9)	850 (70.0)	189.5 (121.9274.0)	80 (6.6)	175.5 (108.5316.3)	10.5	0.005

#### Changes in Tvol with household SIC

Tvol between groups with different household SIC were analyzed. Statistically significant differences among the three groups (*P* = 0.010) were only detected in 1999 but not the other years (Table 5). Pairwise comparisons were performed between the three groups in 1999. The adjusted *P* value < 0.05/3 = 0.017 was considered significant. The adjusted *P*-values were 1.000 between the non-iodized salt group and low-salt iodine group, 0.621 between the non-iodized salt group and high-iodine salt group, and 0.012 between the low-iodine salt group and high-iodine salt group. The results showed that Tvol was higher in the high-iodine salt group than in the low-iodine salt group in 1999.

**Table 5:** Tvol (mL) of 8 to 10 years old children with different household SIC (median; P25, P75)

Year	Non-iodized salt group		Low-iodine salt group		High-iodine salt group		χ2	*P*
	N (%)	Tvol	N (%)	Tvol	N (%)	Tvol		
1999	69 (5.5)	1.2 (0.9,1.5)	215 (17.2)	1.1 (0.9,1.4)	968 (77.3)	1.2 (1.0,1.6)	9.2	0.010
2005	17 (1.4)	2.4 (2.1,2.7)	247 (20.2)	2.4 (2.2,2.8)	959 (38.8)	2.4 (2.2,2.7)	0.7	0.700
2011	95 (7.7)	1.0 (0.8,1.2)	660 (53.5)	1.0 (0.8,1.1)	479 (16.9)	1.0 (0.8,1.2)	0.3	0.865
2014	217 (14.4)	1.9 (1.3,2.4)	1038 (68.7)	1.8 (1,2.5)	255 (16.9)	1.8 (1.1,2.5)	0.8	0.659
2017	283 (23.4)	2.6 (1.8,3.5)	846 (70.0)	2.8 (2,3.7)	80 (6.6)	3.1 (1.6,4.0)	2.1	0.348

## Discussion

ID is the main cause of preventable and irreversible mental retardation in children, and USI is currently implemented in almost all countries worldwide including Shanghai for ID prevention and control [13, 14]. The monitoring results obtained from the Shanghai Municipal Center for Disease Control and Prevention in 2002 showed that the median drinking water iodine concentration was 8.0 μg/L in Shanghai [15]. Despite no prevalence of IDD, the results revealed that ID was prevalent in Shanghai, which justified for the use of iodized salt.

Adequate dietary iodine intake in children is essential for optimal physical and neurological development. According to the standards established by WHO, UNICEF, and ICCIDD [16], 8 to 10 years old children in Shanghai had a iodine intake above the requirement in 1997 and 1999. In 2000, the standard household SIC in Shanghai was appropriately lower, from 20 to 60 mg/kg to 20–50 mg/kg. The average household SIC dropped from 43.8 mg/kg in 1999 to 32.9 mg/kg in 2005. Since 2005, the MUI of children aged 8 to 10 years (171.4–198.1 μg/L) was adequate in Shanghai. Analysis of the changes in MUI among children aged 8 to 10 years by age and sex showed no differences, as previously reported [17]. As of May 7th, 2018, 108 countries have established legislations for salt iodization in an attempt to prevent and control for IDD. However, some countries including the Russian Federation, France, and the UK fail to systematically use iodized salt or other forms of iodine fortification strategies to prevent IDD [18]. Iodine intake in these countries remains a concern. ID has become a public health problem in the UK; some surveys have shown that girls > 15 years of age and pregnant women have insufficient dietary iodine intake [19, 20]. Additionally, national surveys revealed that the general population and pregnant women in Russian Federation and pregnant women in France have insufficient iodine intake [21]. According to the data obtained from the Global Iodine Network in 2017, 143 countries have conducted surveys on the iodine nutritional status of school-age children. Based on the results, MUI values of school-age children in 112 countries were in the appropriate range, accounting for 78.3%. Sixty-nine countries have conducted surveys on the iodine nutritional status of pregnant women. However, only 23 countries (33.3%) reported adequate iodine intake by pregnant women [21].

Even though the average household SIC dropped by nearly 44.5% from 1997 to 2017 and the household coverage with iodized salt decreased from 94.6% in 1997 to 76.5% in 2007, MUI is maintained at approximately 200 μg/L. Goiter rate was consistently low; the maximum goiter rate was 3.07%, lower than the WHO standard for IDD [22]. Our findings revealed no significant differences in MUI among the three household SIC groups since 2005. Consequently, the choice of iodized salt has little effects on UIC of children aged 8–10 years. Similar results were obtained for MTvol. Our findings provide a false sense of the necessity of USI, because iodized salt have little effect on iodine status of Shanghai residents. However, our results may have been affected by the economic level and dietary habits of individuals. Shanghai has usually been the economic center of China. Studies have reported that Shanghai residents consume more animal protein especially milk [23–25], which is rich in casein. Inadequate animal protein intake, which affects the ability of the thyroid glands to absorb and utilize the trace element, is associated with a decline in iodine excretion [26]. A study on the effect of insufficient protein intake on Tvol in ID-prone areas revealed that both iodine deficiency and inadequate protein intake can lead to goiter [27]. In the 1950s, researchers discovered that casein protects against goiter which might explained why the household SIC nearly has no effect on MTvol [28].

With increasing economic and social development, Chinese residents have been dinning out considerable more. Similarly, the consumption of pre-packaged foods has increased. China has implemented the nationwide policy that pre-packaged and prepared-away-from-home meals (e.g., at restaurants and school cafeterias) should incorporate iodized salt. A dietary nutrition survey conducted in Shanghai from 2012 to 2014 showed that the prevalence of dining out ranged between 31.8 and 55.1% in adults [29]. In Shanghai, almost all children from primary and secondary schools have lunch at the school cafeteria. In the past 20 years, with the gradual development of food sales from the traditional food stores to the modern shopping systems such as the chain supermarkets and online shopping, there has been a higher consumption of pre-packaged foods and meals [30].

The reduction of household SIC should not be a concern. On the one hand, the consumption of more animal protein by Shanghai residents will improve the utilization rate of iodine by thyroid glands. On the other hand, even though household SIC is lower, the iodine content of pre-packaged and prepared-away-from-home meals cannot be ignored.

China implemented the salt industry reform system in 2017. Regional restrictions were abolished on the production and marketing of salt, and the salt market was completely established. Non-iodized salt can no longer be sold in designated places, and can be purchased at will on mainstream e-commerce platforms, which may have some impact on the iodine nutritional status of Shanghai residents.

Salt reduction strategies are implemented worldwide to prevent cardiovascular disease. In a report by the Global Action Plan for the Prevention and Control of Non-communicable Diseases (2013–2020), WHO has recommended a 30% reduction in salt consumption worldwide [31]. While this recommendation may be inconsistent with the current salt iodization programs, these two important public health policies can be implemented simultaneously by ensuring that iodized salt has a high penetration rate in food production and adjusting iodized salt fortification levels [32]. At the same time, the monitoring of iodine nutritional status in Shanghai is carried out every year since 2016; therefore, salt reduction strategies will not have a great impact on Shanghai residents.

This is the first study that integrates the results of iodine deficiency monitoring in Shanghai in the last 20 years and analyzes relevant influencing factors. Previous studies have focused only on MUI and the relationship between urinary iodine and thyroid volume. This study analyzed the differences in urinary iodine levels and thyroid volume with different household SIC to provide a basis for the implementation of iodized salt policies.

This study also comes with some limitations. One is related to thyroidal morphology. Goiter is divided into three types, namely diffuse, nodular and mixed, but all data acquired so far only included diffuse goiter. Shanghai has a lowest goiter rate----0.08% in 2005 and 2011, but these data did not include nodular goiter which is a more severe type of goiter. The second limitation is related to thyroidal function. Some functional indicators could have been measured in addition to thyroid volume, for examples, free thyroxine (FT4) and thyrotropin, which are used to diagnose not only subclinical hypothyroidism and overt hypothyroidism, but also isolated hypothyroxinemia and hyperthyroxinemia. Our findings did not take these indicators into consideration.

## Conclusions

Based on our findings, the iodine status of Shanghai residents is appropriate, and household SIC has little effect on the iodine status of residents. Future studies should analyze dietary sources of iodine, especially of prepared-away-from home and pre-packaged food or meals, to help inform accurate prevention and control strategies for ID. These findings underline the importance of regular monitoring of iodine intake, especially with regard to salt consumption and standard household SIC.

## Data Availability

Please contact author for data requests.

## Abbreviations

ICCIDD: International Council for Control of Iodine Deficiency Disorders
ID: Iodine deficiency
IDD: Iodine deficiency disorders
MSI: Mean SIC
MTvol: Median Tvol
MUI: Mean UIC
MUI: Median urinary iodine
SCI: Household salt iodine concentration
Tvol: Thyroid volume
UIC: Urinary iodine concentration
UNICEF: United Nations Children’s Fund
USI: Universal salt iodization
WHO: World Health Organization
